# Somatic *VHL *gene alterations in MEN2-associated medullary thyroid carcinoma

**DOI:** 10.1186/1471-2407-6-131

**Published:** 2006-05-17

**Authors:** Christian A Koch, Frederieke M Brouwers, Alexander O Vortmeyer, Andrea Tannapfel, Steven K Libutti, Zhengping Zhuang, Karel Pacak, Hartmut PH Neumann, Ralf Paschke

**Affiliations:** 1 Division of Endocrinology and Nephrology, University of Leipzig, Philipp-Rosenthalstr. 27, 04103 Leipzig, Germany; 2 Division of Endocrinology, University of Mississippi Medical Center, 2500 N State Str, Jackson, MS 39216, USA; 3 Reproductive Biology and Medicine Branch, National Institute of Child Health and Human Development, National Institutes of Health, Center Drive, Building 10, Bethesda, MD 20892, USA; 4 Surgical Neurology Branch, National Institute of Neurological Disorders and Stroke, National Institutes of Health, Center Drive, Building 10, Rm 5D37, Bethesda, MD 20892, USA; 5 Institute of Pathology, Ruhr-Universität Bochum an den BG Kliniken Bergmannsheil, Bürkle-de-la-Camp-Platz 1, 44 789 Bochum, Germany; 6 Surgery Branch, Center for Cancer Research, National Cancer Institute, 10 Center Drive, Room 4W-5940, Bethesda, MD 20892, USA; 7 Division of Nephrology and Hypertension, Albert-Ludwigs-Universitaet of Freiburg, Hugstetter Str. 55, 79106 Freiburg, Germany

## Abstract

**Background:**

Germline mutations in *RET *are responsible for multiple endocrine neoplasia type 2 (MEN2), an autosomal dominantly inherited cancer syndrome that is characterized by medullary thyroid carcinoma (MTC), pheochromocytoma, and parathyroid hyperplasia/adenoma. Recent studies suggest a "second hit" mechanism resulting in amplification of mutant *RET*. Somatic *VHL *gene alterations are implicated in the pathogenesis of MEN2 pheochromocytomas. We hypothesized that somatic *VHL *gene alterations are also important in the pathogenesis of MEN2-associated MTC.

**Methods:**

We analyzed 6 MTCs and 1 C-cell hyperplasia (CCH) specimen from 7 patients with MEN2A and *RET *germline mutations in codons 609, 618, 620, or 634, using microdissection, microsatellite analysis, phosphorimage densitometry, and *VHL *mutation analysis.

**Results:**

First, we searched for allelic imbalance between mutant and wild-type *RET *by using the polymorphic markers D10S677, D10S1239, and RET on thyroid tissue from these patients. Evidence for *RET *amplification by this technique could be demonstrated in 3 of 6 MTCs. We then performed LOH analysis using D3S1038 and D3S1110 which map to the *VHL *gene locus at 3p25/26. *VHL *gene deletion was present in 3 MTCs. These 3 MTCs also had an allelic imbalance between mutant and wild-type *RET*. Mutation analysis of the VHL gene showed a somatic frameshift mutation in 1 MTC that also demonstrated LOH at 3p25/26. In the 2 other MTCs with allelic imbalance of *RET *and somatic *VHL *gene deletion, no somatic *VHL *mutation could be detected. The CCH specimen did neither reveal *RET *imbalance nor somatic *VHL *gene alterations.

**Conclusion:**

These data suggest that a *RET *germline mutation is necessary for development of CCH, that allelic imbalance between mutant and wild-type *RET *may set off tumorigenesis, and that somatic *VHL *gene alterations may not play a major role in tumorigenesis of MEN2A-associated MTC.

## Background

Medullary thyroid carcinoma (MTC) develops from the parafollicular C cells in the thyroid gland and occurs sporadically as well as in the hereditary syndrome multiple endocrine neoplasia type 2 (MEN2). The gene responsible for MEN2 is the RET protooncogene, located at chromosome 10q11.2 [1,2]. Patients with germline mutations in *RET *invariably develop MTC, although there are some patients with *RET *germline mutations who have lived beyond age 70 without MTC [3,4]. Therefore, patients with germline mutations in *RET *usually are divided into different risk groups (low, intermediate, and high risk for developing MTC). So-called low-risk or level I mutations include those at codons 609, 768, 790, 791, 804, and 891 [1,5-8]. Hyperplasia of the parafollicular C cells has been regarded as the precursor lesion for MTC [9,10]. Accumulating evidence suggests that a 'second hit' or additional genetic events may be required to set off tumorigenesis in susceptible cells of patients with *RET *germline mutations. An allelic imbalance between mutant and wild-type *RET *may represent the decisive step of tumorigenesis for MTC and pheochromocytoma [11-15]. Somatic *VHL *gene alterations are implicated in the pathogenesis of MEN2-associated pheochromocytomas, possibly through accumulation of RET protein [16]. Therefore, we hypothesized that somatic *VHL *gene alterations may also play a role in the pathogenesis of MEN2-associated MTC.

## Methods

### Patients and tissues

Seven patients with MEN2A and *RET *germline mutations in codons 609 (4 patients, Leipzig), 618 (1 patient, St. Louis), 620 (1 patient, St. Louis), or 634 (1 patient, NIH) underwent total thyroidectomy at the Washington University in St. Louis, MO, the National Institutes of Health in Bethesda, and the University of Leipzig, Germany. In 6 patients, MTC was diagnosed; 1 patient had C cell hyperplasia (Tables 1 and 2). None of the patients had clinical evidence for VHL syndrome and was analyzed for germline mutations in the VHL gene.

Two specimens (cases 1 and 2 in Table 1) were previously analyzed for imbalance of the mutant and wild-type *RET *allele (see Ref. [12]).

Frozen or paraffin-embedded tissue was microdissected and analyzed for loss of heterozygosity (LOH) by polymorphic markers mapping to the VHL gene locus, as previously described [16].

Tissue was obtained from these 7 patients under an Internal Review Board (IRB)-approved protocol at the National Institutes of Health. All of them had MEN 2A with a germline mutation in *RET*. Blood was drawn for DNA extraction. Thyroid specimens including MTC were removed at the time of surgery and frozen at -80°C. DNA was extracted from lymphoblasts and tumor tissue by standard methods.

Six-micron sections were obtained from frozen tumor and briefly stained with hematoxylin and eosin (H & E). If no frozen tumor was available, paraffin-embedded tissue was prepared for microdissection. Under direct light microscopic visualization using a 30-gauge needle, a modified microdissection procedure was performed, as previously described [17]. In all cases, we obtained also samples of nontumor control tissue from the same slides.

### Allelic imbalance of the RET gene locus and loss of heterozygosity of the VHL gene locus

We performed imbalance analyses of *RET*, using polymorphic markers/primers D10S677, D10S1239, and RET for the RET locus, and loss of heterozygosity studies using markers D3S1038 and D3S1110 which map to the VHL gene locus 3p25/26 in the presence of [alpha-^32^P] dCTP (0.1 μCi/μl) (Dupont). PCR conditions using AmpliTaq Gold DNA polymerase (Perkin Elmer Roche) in a Hybaid Omnigene thermal cycler were as follows: initial denaturation at 95°C for 10 min, then 35 cycles, each with 1 min of denaturation at 95°C, 1 min of annealing at 55°C for *RET *markers, at 60°C for *VHL *markers, and 1 min of extension at 72°C; PCR was completed with a final extension at 72°C for 10 min. The amplicons were resolved on a 6% polyacrylamide gel. Gels were dried and exposed to Kodak XAR film. All PCR reactions were performed in triplicate.

### VHL gene mutation analysis

Exons 1–3 of the *VHL *gene were amplified from genomic DNA using polymerase chain reaction conditions described elsewhere [18]. Mutation scanning by conformation sensitive gel electrophoresis (CSGE) was performed on the PCR products as described by Ganguly *et al*. [19]. DNA sequence analysis was performed using a cycle sequencing kit with dye-labeled terminators (PE Advanced Biosystems, Inc., Foster City, CA). Sequences were analyzed on an ABI 377 automated DNA sequencer.

## Results

**Figure 1:**
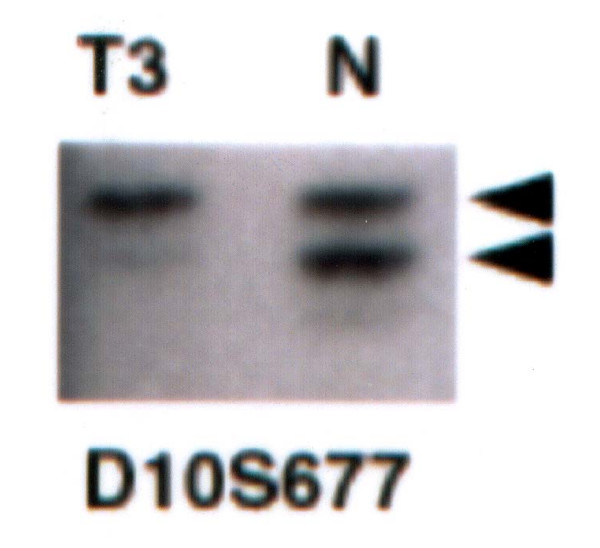
**Allelic imbalance of mutant and wild-type *RET *in one representative case (case 3, Table 1) of MEN2A-associated medullary thyroid carcinoma**. T3, tumor from case 3; N, normal tissue; D10S677, polymorphic marker. The arrows indicate the 2 *RET *alleles.

**Figure 2:**
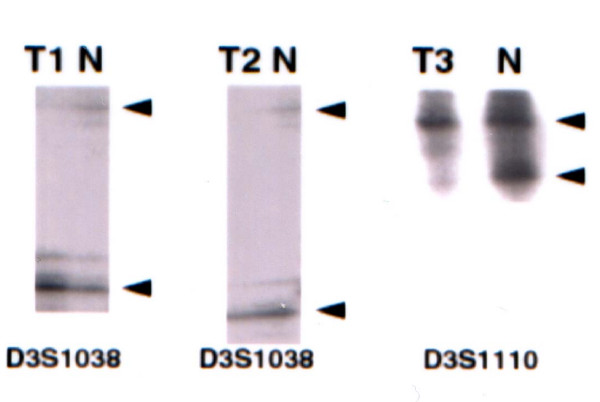
**LOH analysis of the *VHL *gene locus of 3 cases of MEN 2A-related MTCs with marker D3S1038 or D3S1110**. All cases show loss of one allele in microdissected tumor tissue (T), whereas heterozygosity is retained in normal non-neoplastic tissue (N). Arrows indicate the 2 *VHL *alleles.

We studied 6 medullary thyroid carcinomas and 1 C-cell hyperplasia specimen from 7 patients with MEN 2A and known *RET *germline mutations for allelic imbalance at the RET locus and for somatic genetic alterations at the VHL gene locus at 3p25/26 including LOH and mutations (Table 1). Allelic imbalance between the mutant and wild-type *RET *allele could be demonstrated in 3 MTCs (Fig. 1). The same 3 tumors also had LOH of the VHL gene locus (Fig. 2).

**Figure 3:**
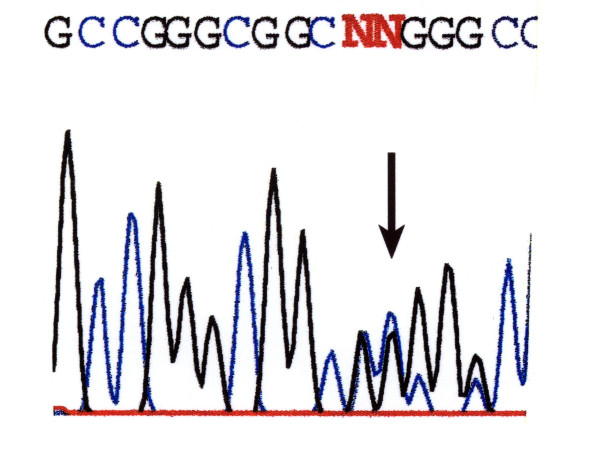
**Somatic mutation of the VHL gene in MEN2-associated MTC**. Portion of the sequence of exon 1 from **case 1 **demonstrating a deletion of a single base (del C) at nucleotide 389 of the VHL gene in one allele at the position indicated by the arrow. This mutation would result in a shift in the translational frame of the encoded mRNA from codon 59 to a premature stop codon at position 66. Exons 1–3 of the VHL gene were amplified from genomic DNA using polymerase chain reaction conditions described elsewhere (Stolle *et al*.,1998). Mutation scanning by conformation sensitive gel electrophoresis (CSGE) was performed on the PCR products obtained using PCR conditions described by Ganguly *et al*., 1993. DNA sequence analysis was performed using a cycle sequencing kit with dye-labeled terminators (PE Advanced Biosystems, Inc., Foster City, CA). Sequences were analyzed on an ABI 377 automated DNA sequencer.

**Figure 4:**
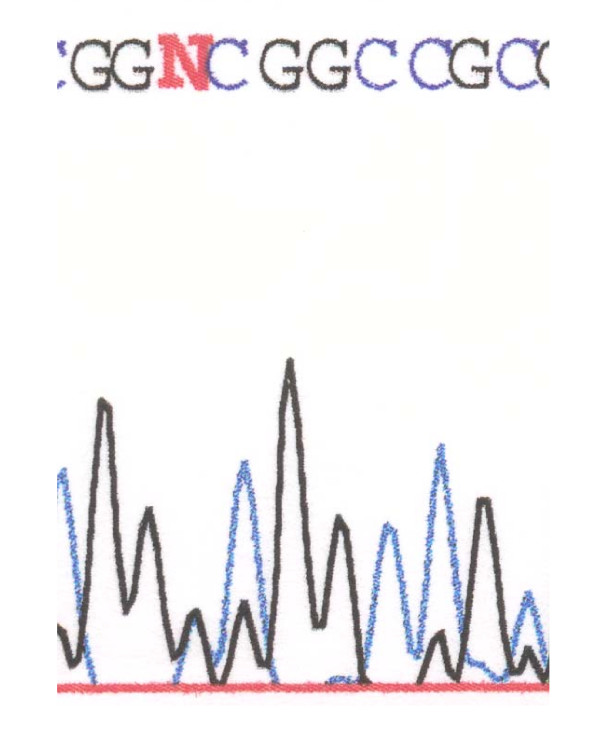
Normal sequence of the VHL gene, portion of exon 1, compared to mutant sequence in figure 3.

To further investigate whether inactivation of the VHL tumor suppressor gene plays a role in tumorigenesis of MEN 2A-related MTC, we performed mutation analysis of the VHL gene and found mutations in at least 1 of the 7 C- cell specimens (Figs. 3 and 4, Table 1). Mutation analysis of the VHL gene showed a frameshift mutation in 1 MTC. This tumor had simultaneously LOH at the RET and VHL gene locus (case 1 in Table 1). Another tumor (case 2) had LOH at the RET and VHL gene locus but no somatic *VHL *mutation. In cases 3 and 4, the mutation analysis for *VHL *could not be completely performed (exon 1 was analyzed and negative for mutations; exons 2 and 3 could not be analyzed) because of technical problems and no further DNA left. Cases 5 and 6 (MTC) as well as case 7 (CCH) had neither LOH at *RET *or *VHL *nor somatic mutations of *VHL*.

## Discussion

In this study, we show that somatic *VHL *mutation and deletion are involved in tumor progression rather than tumor initiation of MEN 2A-related MTC. In both sporadic and familial forms of MTC, allelic losses at 1p, 3, 4, 9q, 10q, 13q, and 22q have been reported [20-24]. Specific tumor suppressor genes or oncogenes at these chromosomal loci, however, have rarely been analyzed.

In sporadic pheochromocytomas, somatic mutations of *VHL *or *RET *are found in less than 10% of tumors, suggesting a possible role in tumorigenesis, whereas the prevalence and role of such mutations in hereditary pheochromocytomas such as those occurring in MEN 2 is not known. In a recent study, we detected inactivating somatic mutations of *VHL *and LOH at 3p25/26 in several MEN 2A-related pheochromocytomas [16]. In all of these MEN 2A-associated pheochromocytomas as well as in the present study of thyroid C cell specimens, regular wild-type *VHL *alleles were detected in normal control tissue confirming the presence of two wild-type alleles and excluding the presence of VHL disease.

VHL disease consists of a variety of tumors including pheochromocytoma [25,26]. However, MTC is not part of VHL syndrome, although MTC was found (likely coincidentally) in 2 patients with *VHL *germline mutation [27]. Mutations in the *VHL *gene lead to constitutive expression of many hypoxia-inducible genes, in part related to increasing levels of hypoxia-inducible transcription factor (HIF) 1-alpha which in normal cells is rapidly ubiquitinated and degraded [28]. VHL protein, a ubiquitinase adapter, plays a role in cell-cycle control, differentiation, extracellular matrix formation and turnover, and angiogenesis. Therefore, mutations in *VHL *may lead to an absent or reduced VHL protein function with reduced degradation of proteins including RET protein. In addition to other events, RET protein accumulation secondary to absent or reduced VHL protein, may then cause further transformation of selected parafollicular C-cells.

Although a 'second hit' which usually consists of deletion in the wild-type VHL allele, is necessary for initiation of tumor growth in patients with *VHL *germline mutations, it may not be sufficient [26,29,30]. Similarly, in patients with MEN2, a *RET *germline mutation is necessary for formation of MTC and pheochromocytoma, but may not be sufficient, as there are patients who have never developed MTC or pheochromocytoma during their life or developed MTC late in their lifetime [3,4,31-33]. Here, we detected somatic *VHL *gene alterations in 3 of 6 MTCs from MEN2A patients and no somatic *VHL *gene alteration in 1 patient with MEN2A and CCH. In addition, all 3 MTCs with LOH at the VHL gene also had allelic imbalance of *RET*. However, in only one of these MTCs, a somatic *VHL *mutation was found in addition to LOH at the VHL gene. In our single case of CCH, no imbalance between mutant and wild-type *RET *has been detected and no somatic *VHL *gene alteration. Therefore, additional somatic mutational events including the VHL gene may be required to set off tumorigenesis in patients with *RET *germline mutations. CCH foci have heterogeneous DNA deletions including LOH of *TP53, RB1*, and *WT1 *[34]. Polymorphisms G691S/S904S of *RET *have recently been found to affect the development of MTC and the age at onset of MEN2A in patients with a *RET *germline mutation [35-37]. Elisei et al. [36] found a statistically significant higher allelic frequency of G691S polymorphism in MTCs than that found in normal controls. Cebrian et al. [37] carried out an association study in 135 sporadic MTC patients and 533 controls and discovered a strong association between the disease and specific haplotypes of *RET*. Somatic *RET *mutations such as M918T in MTC or pheochromocytoma of patients with a *RET *germline mutation appear to represent a phenomenon of tumor progression [38-43]. Chromosomal imbalances in MTC include deletions of chromosomes 1p, 3q26.3-q27, 4, 9q13-q22, 13 q, and 22q [24]. Marsh et al. [24] found loss of whole or partial chromosome 3 including the region of the VHL locus as the predominant imbalance (40% of cases). Biallelic inactivation of *VHL *should lead to loss of VHL protein. Using immunohistochemistry, VHL protein is detectable in thyroid follicles and differentiated tumors derived from follicular epithelium, but is weakly or not detectable in nonneoplastic and neoplastic C cells [44]. Since, in our study, neither allelic imbalance of *RET *nor somatic *VHL *gene alterations occurred in the CCH specimen (presumably the precursor lesion of MTC), we suggest that such somatic *VHL *gene alterations rather play a role in tumor progression than in tumor formation of MEN2-related MTC. This interpretation is based on a single case of CCH and would require confirmation in studies of larger size.

## Conclusion

Tumor formation in these selected MEN 2A-related MTCs may have occurred by first, a *RET *germline mutation, leading to hyperplasia of C cells and second, an amplification event of mutant RET which, however, could not be consistently detected by the methods used by us (polymorphic marker analysis). Subsequent somatic *VHL *gene deletion and mutation in selected C cells may lead to further transformation and tumor progression. However, given the small sample size, it is possible that tumor formation and progression in a significant number of tumors may occur by other mechanisms.

## Abbreviations

MTC – medullary thyroid carcinoma; CCH – c cell hyperplasia; MEN2 – multiple endocrine neoplasia type 2, LOH – loss of heterozygosity; VHL – von Hippel Lindau

## Competing interests

The author(s) declare that they have no competing interests.

## Authors' contributions

CAK designed this study, carried out bench experiments related to this study, interpreted the results, drafted the paper, and finalized the manuscript after input from the other authors.

FBM helped carrying out bench experiments related to this paper. AOV provided microdissection of material used for this study, helped in interpreting the results and drafting the paper by providing critical intellectual input. AT provided material for this paper and helped drafting the paper. SKL provided material for this paper and helped drafting the paper.

ZZ helped in interpreting the results of this study. KP helped providing material for this paper and drafting the paper. HPHN helped in carrying out bench experiments and drafting the paper. RP provided intellectual input in drafting of the paper. All authors read and approved the final version of the manuscript.

## Pre-publication history

The pre-publication history for this paper can be accessed here:



## Figures and Tables

**Table 1:** Somatic *VHL *gene deletion and mutation in MEN 2A-associated medullary thyroid carcinomas/CCH with germline mutations of *RET*

**Case**	***RET *germline Mutation**	**LOH at the *VHL *locus**	**Somatic *VHL *Mutation**	**Allelic imbalance of *RET***
1	Codon 620 TGC/CGC	Yes	del C nt 389 Exon 1	Yes (D10S677)
2	Codon 618 TGC/AGC	Yes	No	Yes (D10S677)
3	Codon 609 TGC/CGC	Yes	N/A	Yes (D10S677)
4	Codon 609 TGC/CGC	No	N/A	No
5	Codon 609 TGC/CGC	No	No	No
6	Codon 609 TGC/CGC	No	No	No
7 (CCH)	Codon 634 TGC/CGC	No	No	No

N/A, not available

**Table 2:** Clinical features

**Case**	***RET *germline Mutation**	**Plasma calcitonin (pg/ml, normal: < 100)**	**Age at thyroidectomy**	**Pheochromo-cytoma/HPT**
1	Codon 620 TGC/CGC	N/A	N/A	N/A
2	Codon 618 TGC/AGC	N/A	N/A	N/A
3	Codon 609 TGC/CGC	118	40 y	No
4	Codon 609 TGC/CGC	120	41 y	No, but HTN
5	Codon 609 TGC/CGC	563	43 y	No, but HTN
6	Codon 609 TGC/CGC	787	44 y	Unilateral pheo
7 (CCH)	Codon 634 TGC/CGC	N/A	25 y	No

N/A, not available; HTN, hypertension
